# Long-term depressive symptoms trajectories following CBT delivered in primary care compared to usual treatment

**DOI:** 10.1017/S0033291724002976

**Published:** 2024-12

**Authors:** Maider Prieto-Vila, César González-Blanch, Rob Saunders, Joshua E. J. Buckman, Roger Muñoz-Navarro, Gabriel Esteller Collado, Sara Barrio-Martínez, Juan A. Moriana, Paloma Ruiz-Rodríguez, María Carpallo-González, Antonio Cano-Vindel

**Affiliations:** 1 Department of Experimental Psychology, Cognitive Processes and Logopedics, Faculty of Psychology, Complutense University of Madrid, Madrid, Spain; 2 Mental Health Centre, University Hospital ‘Marqués de Valdecilla’-IDIVAL, Santander, Cantabria, Spain; 3 CORE Data Lab, Research Department of Clinical, Educational and Health Psychology, University College London, London, UK; 4 iCope – Camden and Islington NHS Talking Therapies for anxiety and depression Services, Camden & Islington NHS Foundation Trust, London, UK; 5 Department of Personality, Assessment and Psychological Treatments, Faculty of Psychology, University of Valencia, Valencia, Spain; 6 Department of Health Psychology, Miguel Hernandez University, Elche, Spain; 7 Department of Psychology, University of Cordoba, Cordoba, Spain; 8 Maimonides Institute for Biomedical Research of Cordoba (IMIBIC)/Reina Sofía University Hospital, Cordoba, Spain; 9 Tres Cantos II Primary Care Centre, Health Service of Madrid, Tres Cantos, Madrid, Spain; 10 Department of Psychology, Faculty of Biomedical and Health Sciences, Universidad Europea de Madrid, Madrid, Spain

**Keywords:** depression, growth mixture modeling, primary care, prognostic factors, treatment trajectories

## Abstract

**Background:**

The course of depression is heterogeneous. The employed treatment is a key element in the impact of the course of depression over the time. However, there is currently a gap of knowledge about the trajectories per treatment and related baseline factors. We aimed to identify trajectories of depressive symptoms and associated baseline characteristics for two treatment arms in a randomized clinical trial: treatment as usual (TAU) or TAU plus transdiagnostic group cognitive behavioral therapy (TAU + TDG-CBT).

**Methods:**

Growth mixture modeling (GMM) was used to identify trajectories of depressive symptoms over 12 months post-treatment. Logistic regression models were used to examine associations between baseline characteristics and trajectory class membership in 483 patients (TAU: 231; TAU + TDG-CBT: 251).

**Results:**

We identified different patterns of symptom change in the randomized groups: two trajectories in TAU (‘improvement’ (71.4%) and ‘no improvement’ (28.6%)), and four trajectories in TAU + TDG-CBT (‘recovery’ (69.8%), ‘late recovery’ (5.95%), ‘chronicity’ (4.77%), and ‘relapse’ (19.44%)). Higher baseline symptom severity and comorbidity were associated with poorer treatment outcomes in both treatment groups and worse emotional regulation strategies were linked to the ‘no improvement trajectory’ in TAU. The TAU + TDG-CBT group demonstrated greater symptom reduction compared to TAU alone.

**Conclusions:**

There is heterogeneity in treatment outcomes. Integration of TDG-CBT with TAU significantly improves symptom reduction compared to TAU alone. Patients with higher baseline severity and comorbidities show poorer outcomes. Identification of trajectories and related factors could assist clinicians in tailoring treatment strategies to optimize outcomes, particularly for patients with a worse prognosis.

## Introduction

Depression is estimated to affect one in 20 adults globally every year (Thornicroft et al., 2017), and it stands as a leading cause of disability worldwide (WHO, 2017). The economic burden of depression is high due to loss of productivity and early retirement (König, König, & Konnopka, 2019; Vieta et al., 2021). Despite its prevalence and impact, the access to evidence-based treatments such as cognitive behavioral therapy (CBT) is not equitable within and between countries (Thornicroft et al., 2017). This had made the integration of evidence-based therapies an international health priority (Patel et al., 2018), especially in primary care settings as it is most common context for treatment (Kovess-Masfety et al., 2007).

The employed treatment is one of the most important prognostic factors to achieve a sustained recovery across the time. However, despite the efficacy of different treatments for depression, there is a proportion of patients that show no response to the treatment or does not achieve a recovery (Cuijpers et al., 2021; Saunders et al., 2019, 2021; Skelton et al., 2023a, 2023b). In addition, for those that achieve a recovery, many will experience relapses over time (Prieto-Vila, Estupiñá, & Cano-Vindel, 2021; Saunders et al., 2021). CBT, in different formats (individual, group, or guided self-help) has been shown to be more effective than treatment as usual (TAU), at short and long-term (Cuijpers et al., 2021; Santoft et al., 2019) and psychological therapies are preferred by patients over pharmacotherapy (McHugh, Whitton, Peckham, Welge, & Otto, 2013), despite pharmacotherapy being the usual treatment in primary care (Watts, Turnell, Kladnitski, Newby, & Andrews, 2015). Previous study modeling the trajectories of PsicAP clinical trial, which is a large RCT with repeated measures in the Spanish primary care context, had found that the addition of seven session of transdiagnostic group CBT (TDG-CBT) to TAU increases the likelihood of recovery in comparison to TAU alone where the likelihood following the trajectories of late recovery, relapse, or chronicity was higher (Prieto-Vila et al., 2024).

Baseline factors such as higher depression severity, comorbidity (e.g. anxiety, panic, or somatic symptoms), ADM consumption, sleeping difficulties, and presence of suicidal thoughts, are also linked with a worse long-term prognosis regardless of the employed treatment (Buckman et al., 2021; Buckman, Saunders, Fearon, Leibowitz, & Pilling, 2019; O'Driscoll et al., 2021; Prieto-Vila et al., 2024). Similar results were found during CBT in IAPT services (Saunders et al., 2019, 2021; Skelton et al., 2023a). Understanding which patients are more likely to benefit from one specific treatment and which patients are not, offers the opportunity to for precision mental health care and better-informed treatment decision making for clinicians and patients alike (Deisenhofer et al., 2024). However, there is still limited knowledge of prognosis with particular treatments, and few studies leveraging large randomized controlled trial data to elucidate means of identifying differential treatment response to inform decision making prior to treatment being initiated.

Therefore, the present study aimed to (1) examine the trajectory of depressive symptoms changes up to 12 months post-treatment, separately for TAU with and without TDG-CBT on a PsicAP clinical trial; and (2) identify associations between baseline characteristics and trajectories for each treatment.

## Methods

### Design and participants

Data from the PsicAP longitudinal randomized clinical trial, conducted in 22 primary care centers across Spain, were used (Cano-Vindel et al., 2022). 1061 patients were randomly allocated (1:1) to TAU (*n* = 534) or to TAU + TDG-CBT (*n* = 527). Inclusion criteria were being aged from 18 to 65 years and scoring above the cut-off points on one or more of the screening scales for depression, generalized anxiety, or somatoform disorder (PHQ-9 ≥ 10; GAD-7 ≥ 10; PHQ-15 ≥ 10, respectively). Exclusion criteria were severe symptoms of depression (PHQ-9 ≥ 24); high level of disability (SDS ≥ 26); recent suicidal behavior; receiving another psychological treatment; having difficulties understanding Spanish; having a diagnosis of substance dependence disorder; or a severe mental illness confirmed by clinical interview with a clinical psychologist (i.e. personality disorders, eating disorders, bipolar disorder, or a psychotic condition).

For the current study, we selected patients scoring at least ‘mild’ depression severity at baseline (PHQ-9 ≥ 5) and who had completed pretreatment, posttreatment and at least one follow-up assessment (3, 6, or 12 months) to achieve the necessary data for modelling the trajectories of depression symptoms.

### Interventions

The TAU intervention consisted of regular consultations with the treating GP. In general, these treatments involved the prescription of anxiolytics, antidepressants, and/or informal counseling.

The TAU + TD-GCBT consisted of TAU treatment plus the addition of seven 90-min therapy sessions held over a 12–14-week period in small groups (8–10 patients) at the primary care center. Sessions were led by a senior clinical psychologist, who received a detailed, session-by-session outline of the treatment. The therapeutic approach was based on the transdiagnostic approach to emotional disorders, which assumes that most emotional disorders share several common factors, and that the onset and maintenance of emotional disorders are due to dysregulated cognitive-behavioral emotion regulation strategies (Aldao & Nolen-Hoeksema, 2010; Hofmann & Barlow, 2014).

Preliminary analyses were conducted to examinate if there are baseline differences between patients per treatment in this analytical sample. No statistically significant differences were found, except on metacognitive beliefs were the TAU + TDG-CBT group had higher scores (*M*: 16.64; s.d.: 3.96) in comparison to the TAU (*M*: 15.84; s.d.: 4.03), (*p* = 0.029). This difference may be spurious, a product of multiple comparisons (see Table 1).

**Table 1.** Descriptive statistics total sample and per treatment

	Total (*N* = 483)	TAU (*n* = 231)	TAU + TDG-CBT (*n* = 252)	*p* Value (*t* test)	Statistical power
	Mean (s.d.)	Mean (s.d.)	Mean (s.d.)		
Age	44.69 (11.25)	45 (11.72)	44.4 (10.81)	0.559	1
PHQ-9	14.13 (4.96)	14.12 (4.97)	14.14 (4.95)	0.962	1
PHQ-15	14.43 (4.62)	14.62 (4.45)	14.27 (4.77)	0.402	1
GAD-7	12.67 (4.41)	12.47 (4.35)	12.86 (4.47)	0.333	0.98
SDS	23.87 (9.29)	23.48 (9.47)	24.23 (9.11)	0.374	0.66
WHOQOOL-BREF	2.88 (0.79)	2.89 (0.78)	2.87 (0.81)	0.796	0.9
PSWQ-A	29.99 (6.68)	30.15 (6.5)	29.85 (6.84)	0.629	0.93
RRS brooding	13.36 (3.56)	13.21 (3.55)	13.49 (3.57)	0.402	0.98
IACTA brief	8.37 (5.18)	7.96 (5.13)	8.75 (5.21)	0.098	0.32
ERQ suppression	15. 51 (5.9)	15.24 (6.04)	15.74 (5.76)	0.349	0.28
ERQ reinterpretation	25.35 (6.87)	24.77 (6.76)	25.89 (6.96)	0.075	0.37
MCQ negative beliefs	16.26 (4.01)	15.84 (4.03)	16.64 (3.96)	0.029	0.44
	*n* (%)	*n* (%)	*n* (%)	*p* value (χ^2^)	
Gender				0.157	
Female	393 (81.4)	194 (84)	199 (79)		
Male	90 (18.6)	37 (16)	53 (21)		
Marital status				0.427	
With partner	339 (70.2)	158 (68.4)	181 (71.8)		
Without partner	144 (29.8)	73 (31.6)	71 (28.2)		
Educational level				0.190	
Basic studies	345 (71.4)	172 (74.5)	173 (68.7)		
High studies	138 (28.6)	59 (25.5)	79 (31.3)		
Employment status				0.648	
Employed	255 (52.8)	119 (51.5)	136 (54)		
Unemployed	228 (47.2)	112 (48.5)	116 (46)		
Antidepressant use				0.206	
No	364 (75.4)	168 (72.7)	196 (77.8)		
Yes	119 (24.6)	63 (27.3)	56 (22.2)		
Anxiolytic use				0.223	
No	302 (62.5)	151 (65.4)	151 (59.9)		
Yes	181 (37.5)	80 (34.6)	101 (40.1)		
PHQ-PD				0.919	
Absence	351 (72.7)	72.3 (72.3)	184 (73)		
Presence	132 (27.3)	64 (27.7)	68 (27)		

s.d., standard deviation; PHQ-9, patient health questionnaire-9; PHQ-15, patient health questionnaire-15; GAD-7, generalized anxiety disorder-7; PHQ-PD, patient health questionnaire-panic disorder; WHOQOL, World Health Organization Quality of Life; SDS, Sheehan Disability Scale; PSWQ, Penn State Worry Questionnaire; RRS, Rumination Response Scale; IACTA, Inventory of Cognitive Activity in Anxiety Disorders; ERQ, emotional regulation questionnaire; MCQ, metacognition questionnaire; TAU, treatment as usual; TDG-CBT, transdiagnostic group cognitive-behavioral therapy.

### Measures

Symptoms of depression (PHQ-9; Diez-Quevedo, Rangil, Sanchez-Planell, Kroenke, & Spitzer, 2001; Spitzer, Kroenke, & Williams, 1999). The scale consists of 9 items on a Likert scale from 0 (not at all) to 3 (nearly every day). Total scores range from 0 to 27. Interpretation: 0–4 none-minimal depression; 5–9 mild/subthreshold depression; 10–14: moderate depression; 15–19: moderately severe; 20–27: severe depression. Internal consistency: *α* = 0.75.

Symptoms of Anxiety (GAD-7; García-Campayo et al., 2010; Spitzer, Kroenke, Williams, & Löwe, 2006), consists of 7 items on a Likert scale from 0 (not at all) to 3 (nearly every day). Total scores range from 0 to 21. Interpretation: 0–4: none-minimal anxiety; 5–9: mild anxiety; 10–14: moderate anxiety; 15–21: severe anxiety. Internal consistency *α* = 0.79.

Symptoms of somatization (PHQ-15; Cano-García et al., 2020; Kroenke, Spitzer, & Williams, 2002). It is 15 items on a Likert scale from 0 (not bothered) to 2 (bothered a lot). The total score ranges from 0 to 30. Interpretation: 0–4 none-minimal somatization; 5–9 mild/subthreshold somatization; 10–14: moderate somatization; 15–30: severe somatization. Internal consistency: *α* = 0.68.

Symptoms of panic disorder (PHQ-PD; Muñoz-Navarro et al., 2016; Spitzer et al., 1999). It is 15 items dichotomic (yes/no) scale used to determine the presence or absence of panic disorder employing the DSM algorithm. Presence: the first item must be ‘yes’ and at least one of the next 3 items plus 4 of the somatic symptoms.

Worry (PSQW-A; Meyer, Miller, Metzger, & Borkovec, 1990; Sandin, Chorot, Valiente, & Lostao, 2009). It is an 8-item based questionnaire to measure worry, with a maximum score of 40. Each item is a Likert scale from 1 (it is not typical in me) to 5 (it is very typical in me). Internal consistency: *α* = 0.89.

Rumination brooding subscale (RRS-B; Hervás, 2008; Nolen-Hoeksema & Morrow, 1991). It is 5-items subscale with a Likert-type response scale from 1 (almost never) to 4 (almost always). Internal consistency: *α* = 0.76.

Metacognition negative believes subscale (MCQ-NB; Ramos-Cejudo, Salguero, & Cano-Vindel, 2013; Wells & Cartwright-Hatton, 2004). It is a 5-item subscale of MCQ-30 developed to assess the negative beliefs about uncontrollability and danger, ranging from 5 to 24 measured by Likert scale 1 (totally disagree) to 4 (totally agree). Internal consistency: *α* = 0.80.

Emotional regulation (ERQ; Cabello, Salguero, Fernández-Berrocal, & Gross, 2013; Gross & John, 2003). It is a 10-item scale to assess by two subscales adaptative (ERQ-R, cognitive reappraisal) and maladaptive (ERQ-S, expressive suppression) emotion regulation strategies. Responses are given by a Likert scale from 1 (strongly disagree) to 7 (strongly agree). Internal consistency: *α* = 0.75.

Attentional and Cognitive biases (IACTA-PB; Muñoz-Navarro et al., 2021). It is a 5-item scale to measure attentional and cognitive biases by a Likert scale from 0 (almost never) to 4 (almost always) with maximum punctuation of 20. Internal consistency: *α* = 0.86.

Quality of life (WHOQOL-Bref; Lucas-Carrasco, 2012). It is a 26-item scale to assess the quality-of-life domains (physical, psychological, and health and social). The scale is ranging from 26 to 130 by Likert scale which ranges from 1 (very bad) to 5 (very good). Internal consistency: *α* = 0.86.

Disability (SDS; Bobes et al., 1999; Sheehan, Harnett-Sheehan, & Raj, 1996). It is a 5-item Likert scale from 0 (not at all) to 10 (extremely) to assesses the interference of their symptoms in five daily domains (work, social, and family and stress and social support). 1, 4, and 7 are the cut points for mild, moderate, and high disability, respectively. Internal consistency: *α* = 0.71.

Demographics: self-reported gender, age, marital status (with or without partner), educational level (basic studies, ≤ secondary education and high studies, ≥ university degree) and employment situation (employed or unemployed).

Treatment: treatment as usual, or treatment as usual + transdiagnostic group cognitive behavioral therapy.

Psychiatric medication: currently taking antidepressants or anxiolytics (yes/no).

### Data analysis

Growth Mixture Modeling (GMM; Muthén & Muthén, 2000) was used to identify distinct subgroups of patients who demonstrate similar patterns of responses over time. To run GMM the PHQ-9 scores at pretreatment, posttreatment, and at least one follow-up timepoints (3, 6, and 12 months) were used.

To identify latent classes, GMM analysis was performed modelling up to six classes. These were fitted with linear, quadratic, and log-linear slopes to find the best fitting form. To determine the optimal number of classes, each model (*k*) was compared to the previous model (*k* − 1) on the following recommended model fit statistics: the Vuong-Lo-Medell-Rubin Likelihood Ratio Test (VLMR-LRT) where a *p* value of <0.05 indicates the *k* model is a better fit for the data than the *k* − 1 model, the Akaike Information Criterion (AIC) and the Bayesian Information Criterion (BIC), for which the lowest value between models indicates better fit (Vrieze, 2012). The entropy value of each model was considered, where scores range from 0 to 1 to indicate the accuracy of classification into latent classes, a value ≥0.8 indicates high accuracy in where at least the 80% of the time individuals were correctly classified in latent classes, between 0.8 and 0.40 indicates medium accuracy and ≤0.4 low accuracy (Clark & Muthén, 2009). Following recommendations (Jung & Wickrama, 2008; Muthén & Muthén, 2000; van de Schoot, Sijbrandij, Winter, Depaoli, & Vermunt, 2017) we fixed the variance of the slope to zero, so that the trajectory classes can only differ in the intercept of the starting score. We compared this with a simpler model where the variance in each intercept and slope could have a non-zero value to select the best fit model. This specification has successfully been used in previous studies with patients with depression symptoms in primary care (e.g. Prieto-Vila et al., 2024; Skelton et al., 2023a, 2023b).

In scenarios where the fit indices provided conflicting results, the BIC was considered the primary metric, following recommendations (Nylund, Asparouhov, & Muthén, 2008). GMM analyses were conducted separately for each treatment using Mplus version 8.7 (Muthén & Muthén, 2017). Missing PHQ-9 data were handled using full information maximum likelihood (FIML) and the expectation maximization (EM) algorithm in Mplus (Dempster, Laird, & Rubin, 1977).

### Association of patient and treatment characteristics with trajectory class

Associations between measured baseline variables (see Table 1 for list of variables) and trajectory class membership were tested using binomial logistic regression for TAU, since two trajectories were identified, and multinomial logistic regression for TAU + TDG-CBT, as four trajectories were identified. The variables entered into the multivariable regression models were those with *p* values less than 0.05 in univariable analyses (ANOVA and *t* test for continuous variables, and χ^2^ test for categorical variables), between the trajectories in each treatment. These analyses were conducted using SPSS version 27 (IBM Corp., 2020).

## Results

### Descriptive statistics

For the current study 483 patients from the PsicAP trial met inclusion criteria. Of those 483 (100%) had completed pretreatment and posttreatment assessments, 414 (85.71%) completed the 3 months, 361 (74.74%) completed the 6 months, and 316 (65.42%) completed the 12 months follow-up assessments. A total of 231 patients were randomized to TAU and 252 to TAU + TDG-CBT. Detailed patients baseline characteristics are presented in Table 1 and a flow-chat of the sample is detailed on online Supplementary Fig. S1.

### Trajectories of depressive symptoms per treatment

**Figure 1.**
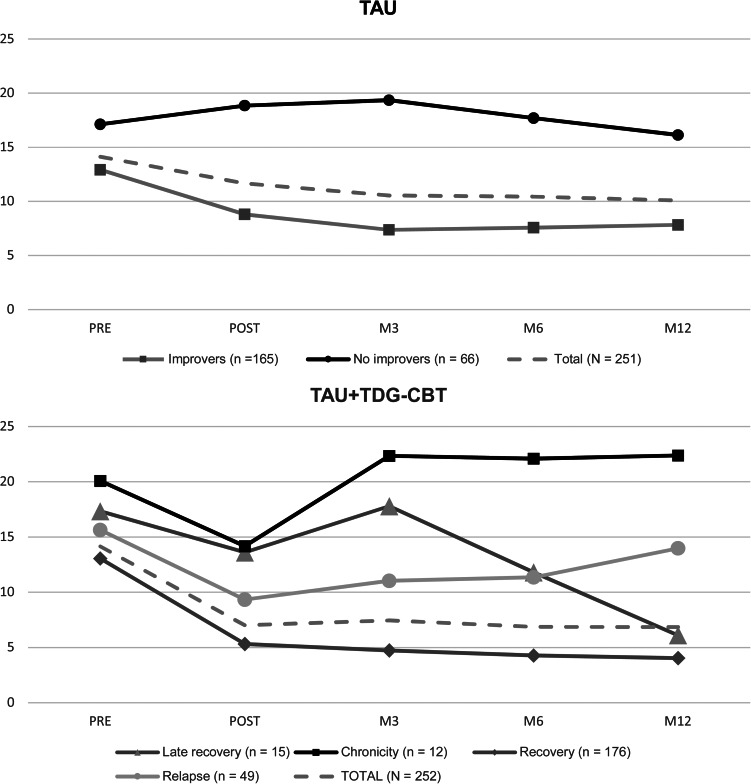
*Depression* trajectories per treatment.

For both treatment groups, a model with a quadratic slope and residuals fixed to zero has shown the best fit to the data in comparison to quadratic, linear, linear with, and without residuals fixed to zero or free loading slopes (see Supplementary materials, Tables S1A–E and S2A–E). The optimum class solution for the TAU group was a two-class model and for the TAU + TCG-CBT group was a four-class model (see Table 2). These class solutions were chosen for each treatment since they had the lowest BIC value and provided a good entropy value (close to high accuracy (0.8)). Additionally, for the TAU group, the two-model solution is better than one model solution according with VLMR-LRT *p* value. Patient class allocation per treatment resulted in the following trajectory groups (see Fig. 1):

**Table 2.** Results of growth mixture modelling analysis

Treatment as usual								
Class solution	Log-likelihood	H0scaling	AIC	BIC	Adj-BIC	VLMR-LRT *p* value	Entropy	Classification (*n* per profile)
**2-class**	**−2931.935**	**1.1489**	**5889.870**	**5934.621**	**5893.419**	**0.0002**	**0.768**	**165/66**
3-class	−2924.393	1.1984	5882.787	5941.308	5887.427	0.3559	0.772	11/62/158
4-class	−2915.584	1.2082	5873.168	5945.459	5878.901	0.2452	0.727	51/29/132/19
5-class	−2910.593	1.0711	5871.185	5957.246	5878.010	0.1547	0.746	2/53/17/125/34
6-class	−2907.352	0.9825	5872.704	5972.535	5880.621	0.1646	0.731	11/116/53/30/3/18
Treatment as usual + transdiagnostic group cognitive behavioral therapy								
Class solution	Log-likelihood	H0scaling	AIC	BIC	Adj-BIC	VLMR-LRT *p* value	Entropy	Classification (% per profile)
2-class	−3201.948	1.7812	6429.896	6475.778	6434.566	0.1943	0.833	35/217
3-class	−3186.959	1.4718	6407.917	6467.918	6414.025	0.1066	0.788	176/13/63
**4-class**	**−3173.847**	**1.3983**	**6389.695**	**6463.813**	**6397.240**	**0.1996**	**0.796**	**15/12/176/49**
5-class	−3164.662	1.3715	6379.324	6467.560	6388.306	0.3905	0.760	16/25/12/126/73
6-class	−3158.765	1.2222	6375.531	6477.884	6385.950	0.4117	0.788	124/75/1/16/25/11

AIC, Akaike information criterion; BIC, Bayesian information criterion; Adj-BIC, sample size-adjusted Bayesian information criterion; VLMR-LRT, Vuong–Lo–Mendell–Rubin likelihood ratio test.

#### Treatment as usual

Class 1 – *improvement* (*n* = 165; 71.4%): characterized by moderate symptoms at baseline (PHQ-9 mean = 12.9; s.d. = 4.7), decrease at posttreatment (PHQ-9 mean = 8.8; s.d. = 4.9) a slight decrease at 3 months follow-up (PHQ-9 mean = 7.37; s.d. = 3.9), and very small increases at 6 months (PHQ-9 mean = 7.57; s.d. = 4.7) and 12 months (PHQ-9 = 7.82; s.d. = 5.3).

Class 2 – *no improvement* (*n* = 66; 28.57%): characterized by showing moderate–severe symptoms at baseline (PHQ-9 mean = 17.5; s.d. = 4.7) a gradual increase at post-treatment (PHQ-9 mean = 18.85; s.d. = 4.4) and 3 months assessments (PHQ-9 mean = 19.35; s.d. = 4) following by a gradual reduction of symptoms at 6 months (PHQ-9 mean = 17.69; s.d. = 5.3) and 12 months (PHQ-9 mean = 16.13; s.d. = 5.9).

#### TAU + TDG-CBT

*Class 1 – recovery* (*n* = 176; 69.84%): characterized by moderate symptoms at baseline (PHQ-9 mean = 13.05; s.d. = 4.6), a pronounced decrease at post-treatment (PHQ-9 mean = 5.31; s.d. = 3.8) and a gradual continuation of symptom reduction at follow-ups: 3 months (PHQ-9 mean = 4.73; s.d. = 3), 6 months (PHQ-9 mean = 4.27; s.d. = 3.3), and 12 months (PHQ-9 = 4.03; s.d. = 3.1).

*Class 2 – late recovery* (*n* = 15; 5.95%): characterized by showing moderate–severe symptoms at baseline (PHQ-9 mean = 17.33; s.d. = 4.7) with a reduction of the symptoms at post-treatment (PHQ-9 mean = 13.6; s.d. = 4.9), pronounced increase at 3-month follow-up assessment (PHQ-9 mean = 17.79; s.d. = 4.3), pronounced symptom reductions at 6 (PHQ-9 mean = 11.8; s.d. = 6.2), and 12-month follow-ups assessment (PHQ-9 mean = 6.1; s.d. = 3.3).

*Class 3 – chronic* (*n* = 12; 4.77%): characterized by showing severe symptoms at baseline (PHQ-9 mean = 20.08; s.d. = 3) following by a pronounced decrease at posttreatment (PHQ-9 mean = 14.17; s.d. = 7.4) and very large increase at 3 months (PHQ-9 mean = 22.33; s.d. = 3.5), which was maintained at similar levels throughout 6 months (PHQ-9 mean = 22.09; s.d. = 2.9) and 12 months (PHQ-9 mean = 22.38; s.d. = 3.3) follow-up assessments.

*Class 4 – relapse* (*n* = 49; 19.44%): characterized by moderate–severe depressive symptoms at baseline (PHQ-9 mean = 15.63; s.d. = 5), a pronounced decrease at posttreatment (PHQ-9 mean = 9.33; s.d. = 4.3), and a gradual increase of symptoms during the follow-up assessments: 3 months (PHQ-9 mean = 11.03; s.d. = 3.3), 6 months (PHQ-9 mean = 11.34; s.d. = 3.2) and 12 months (PHQ-9 mean = 13.97; s.d. = 3.4).

### Associations of baseline variables with trajectory class

#### Treatment as usual

Description about patient's baseline characteristics per trajectory in TAU is detailed in online Supplementary Table S3. The likelihood of being in Class 2-no improvement, relative to Class 1 – improvement was greater in patients with higher baseline scores on the PHQ-9, GAD-7, RRS-brooding subscale, and ERQ-suppression subscale, and lower quality of life scores (Table 3).

**Table 3.** Associations between baseline characteristics and PHQ-9 trajectory class 1 – improvers relative to class 2 – no improvers

Baseline predictor	No improvers OR (95% CI) & *p* value
PHQ-9	1.217 (1.134–1.306), *p* < 0.001
GAD-7	1.143 (1.036–1.262), *p* = 0.008
PHQ-15	1.069 (0.984–1.161), *p* = 0.113
Anhedonia	1.048 (0.667–1.724), *p* = 0.833
Sleeping disturbances	1.114 (0.754–1.647), *p* = 0.588
Suicidal thoughts	
Absence	1.113 (0.581–1.647), *p* = 0.783
Presence	Ref.
SDS	1.003 (0.964–1.045), *p* = 0.867
WHOQOOL	0.429 (0.250–0.738), *p* = 0.002
PSWQ	1.009 (0.945–1.077), *p* = 0.794
ERQ suppression	1.114 (1.05–1.182), *p* < 0.001
RRS brooding	1.136 (1.011–1.28), *p* = 0.033
Antidepressant use	
No	1.851 (0.866–3.956), *p* = 0.112
Yes	Ref.

PHQ-9, Patient Health Questionnaire-9; PHQ-15, Patient Health Questionnaire-15; GAD-7, generalized anxiety disorder-7; WHOQOL, World Health Organization Quality of Life; SDS, Sheehan Disability Scale; PSWQ, Penn State Worry Questionnaire; RRS, Rumination Response Scale; ERQ, Emotional Regulation Questionnaire.

#### TAU + TDG-CBT

Description about patient's baseline characteristics per trajectory in TAU + TDG-CBT is detailed in online Supplementary Table S4. The likelihood of being in Class 2 – late recovery, relative to Class 1 – recovery was higher in patients with higher baselines scores on the PHQ-9 or GAD-7; and for those not taking ADM.

The likelihood of being in trajectory Class 3 – chronicity, compared to Class 1-recovery was higher in those patients that had higher scores on the PHQ-9 or PHQ-15. Higher quality of life scores was associated with a lower likelihood of following the chronicity rather than recovery trajectory.

The likelihood of being in trajectory Class 4-relapse, compared to Class 1 – recovery, was higher in patients with higher PHQ-9 and PHQ-15 scores. See Table 4.

**Table 4.** Associations between baseline characteristics and PHQ-9 trajectory classes 2, 3, and 4 relatives to class 1 (recovery) in TAU + TDG-CBT

Baseline predictor	Late recovery OR (95% CI) & *p* value	Chronicity OR (95% CI) & *p* value	Relapse OR (95% CI) & *p* value
PHQ-9	1.214 (1.08–1.37), *p* < 0.001	1.44 (1.21–1.73), *p* < 0.001	1.12 (1–1.2), *p* < 0.001
GAD-7	1.204 (1–1.449), *p* = 0.049	0.941 (0.71–1.247), *p* = 0.671	1.028 (0.934–1.132), *p* = 0.568
PHQ-15	1.135 (0.998–1.291), *p* = 0.054	1.491 (1.16–1.916), *p* = 0.002	1.104 (1.021–1.193), *p* = 0.013
Anhedonia	0.690 (0.327–1.454), *p* = 0.329	2.567 (0.618–10.67), *p* = 0.195	1.077 (0.718–1.617), *p* = 0.719
Suicidal thoughts			
Absence	0.577 (0.172–1.929), *p* = 0.372	0.176 (0.031–1.016), *p* = 0.052	0.542 (0.267–1.103), *p* = 0.091
Presence	Ref.	Ref.	Ref.
SDS	1.04 (0.964–1.121), *p* = 0.312	0.927 (0.834–1.031), *p* = 0.162	0.989 (0.947–1.033), *p* = 0.613
WHOQOOL	1.204 (0.564–2.573), *p* = 0.631	0.239 (0.062–0.927), *p* = 0.038	0.842 (0.536–1.324), *p* = 0.457
PSWQ	1.036 (0.914–1.174), *p* = 0.583	1.148 (0.934–1.41), *p* = 0.191	1.013 (0.956–1.074), *p* = 0.662
RRS	0.953 (0.773–1.177), *p* = 0.656	1.158 (0.861–1.558), *p* = 0.331	1.057 (0.936–1.193), *p* = 0.373
Antidepressant use			
No	0.149 (0.042–0.536), *p* = 0.004	0.212 (0.043–1.043), *p* = 0.056	0.595 (0.26–1.36), *p* = 0.218
Yes	Ref.	Ref.	Ref.

PHQ-9, Patient Health Questionnaire-9; PHQ-15, Patient Health Questionnaire-15; GAD-7, Generalized Anxiety Disorder-7; WHOQOL, World Health Organization Quality of Life; SDS, Sheehan Disability Scale; PSWQ, Penn State Worry Questionnaire; RRS, Rumination Response Scale.

A logistic regression model was developed to compare Class 2 – late recovery and Class 4 – relapse due the similar intercept (baseline PHQ-9 score) but different trajectories across time using a Class 4 – relapse as a reference. No significant differences were found in baseline characteristics. See online Supplementary Table S5.

## Discussion

### Main results

This study identified different trajectories of change in depressive symptoms during 1-year follow-up in the two treatments arms (TAU and TAU + TDG-CBT) of the PsicAP clinical trial. Baseline variables associated with each trajectory were also identified in primary care patients. The findings showed more heterogeneous course in TAU + TDG-CBT (recovery, late recovery, chronicity, and relapse) than TAU alone (improvement or no improvement).

Previous studies on PsicAP trial have demonstrated that the addition of TDG-CBT to TAU was associated with considerably greater symptom reduction at posttreatment (*p* < 0.001; *d* = −0.58) and at 12 months follow up (*p* < 0.001; *d* = −0.36) compared to TAU alone (Cano-Vindel et al., 2022) and with a greater likelihood of recovery (Prieto-Vila et al., 2024). However, previous studies did not provide information about the heterogeneous course of depressive symptoms across treatments and related factors. This knowledge is crucial to understand which patients could achieve better outcomes per treatment and the degree to which they will improve. In keeping with this, in this study, all trajectories identified in TAU + TDG-CBT group showed a pronounced reduction of symptoms at posttreatment and the ‘recovery’ trajectory was the most prevalent (69.8%) with many patients experiencing a reliable improvement, more than a six points reduction on the PHQ-9, with slight improvement during follow-up assessments. Similar results were found in studies from IAPT during CBT treatment (Saunders et al., 2019; Skelton et al., 2023a). In contrast, in the TAU treatment group, the most prevalent trajectory was ‘improvement’ (71.4%) but the reduction of symptoms at posttreatment was smaller (PHQ-9 from 12.9 to 8.9) than in TAU + TDG-CBT (PHQ-9 from 13.05 to 5.31), underscoring the potential benefits of integrating TDG-CBT into primary care settings.

The other identified trajectory in TAU was one of ‘no improvement’ (28.57%), characterized by high scores with slight fluctuations (decrease or increase) across the endpoints. Trajectories in TAU differed from the trajectories in TAU + TDG-CBT, where the trajectories followed a course of symptom reduction at posttreatment and only the chronicity trajectory (4.77%) and late recovery trajectory (5.95%), those with the lowest prevalence, had no scores under 10 in the PHQ-9 posttreatment (cut-off point). A relapse trajectory (19.44%) was also identified, the mean score of which was close to 10 at posttreatment (9.33) and was characterized by a slight increase of symptoms during follow-ups. The prevalence of these trajectories is lower than those identified in previous studies in primary care, it could be due to the characteristics of the patients in our sample, where patients with PHQ-9 scores of 24 or above were excluded from the trial, whereas in other studies, patients with more severe symptoms were included. It is well evidenced that higher baseline severity is associated with a worse prognosis regardless of treatment type (Buckman et al., 2021). However, a previous study of the PsicAP clinical trial (González-Blanch et al., 2022) investigated the interaction between pre-treatment depressive symptom severity and treatment conditions, finding that all patients with different baseline severity (low/middle/high) benefitted more in TAU + TDG-CBT than in TAU alone at short (posttreatment) and long-term (12 months follow-up). However, it was observed that the benefits were greater for patients with higher baseline severity in depressive symptoms. These results are in line with the findings of the current study in which patients with higher baseline severity in TAU remained at a similar level of severity over time, while patients with high scores at baseline in TAU + TDG-CBT showed a pronounced decrease in symptoms at posttreatment and heterogenous course at 12 months (decrease or increase in symptoms).

Higher symptoms of generalized anxiety in TAU were associated with following the ‘no improvement’ trajectory, while in TAU + TDG-CBT, it was associated with following the ‘late recovery’ trajectory. Higher symptoms on somatization were associated with following either the ‘chronicity’ or ‘relapse’ trajectories in TAU + TDG-CBT. It is noteworthy that the trajectories with worse prognoses (chronicity and relapse) in TAU + TDG-CBT were associated with somatization symptoms. These findings align with the main PsicAP trial where the lower effect size between treatments were found in the PHQ-15 score (Cano-Vindel et al., 2022). Therefore, this could explain the greater likelihood of following chronicity or relapse in patients which received this treatment and had a higher severity of somatization at baseline. In contrast, here we found that a higher severity of generalized anxiety symptoms was associated with following the ‘late recovery’ trajectory, which is also associated with a worse response to psychological treatment on previous research (Buckman et al., 2021; Skelton et al., 2023a). However, it is important to note that the PsicAP clinical trial sample is heterogeneous, and depression may not be the main problem of the patients as the inclusion criteria of the original study was to score 10 or more on symptoms of somatization, generalized anxiety or depression (Cano-Vindel et al., 2022). Therefore, to ensure that patients have at least mild depressive symptoms one of the inclusion criteria for this study was to score 5 or more on the PHQ-9 at baseline. In the line of previous literature about the high comorbidity of the emotional disorders (Hofmann & Barlow, 2014), in the present study, the high correlation and comorbidity between anxiety, depressive and somatic symptoms are notorious, where the average score was more than 10 (cut-off point) at baseline on the main scales in each trajectory in both treatments, where higher punctuation on depression is also a signal of more anxiety or somatic symptoms.

Therefore, it can be expected for that many patients change in one measure, will have impact in other measures too, especially on those patients on TAU + TDG-CBT group, where the treatment approach was transdiagnostic. Moreover, previous study of PsicAP trial about interaction effect between comorbidity (depression and anxiety) and treatment conditions, suggest that the addition of TDG-CBT to TAU leads to better outcomes, especially when comorbid anxiety and depression coexist in patients, compared to TAU alone (González-Blanch et al., 2022).

Previous studies of PsicAP have found that patients receiving TAU + TDG-CBT had significant changes in worry, rumination, metacognitive beliefs, and emotional suppression, and that this was found to mediate the reduction in depressive symptoms. These differences were not observed in patients receiving TAU alone (Barrio-Martínez et al., 2023, 2022). In the present study, the likelihood of following the ‘no improvement’ trajectory in the TAU group was associated with higher rumination and emotional suppression in comparison with following the ‘improvement’ trajectory.

### Strengths and limitations

This study is the first conducted on a large sample of adults in Spanish primary care services aiming to examine the heterogeneous course of depressive symptoms and related factors per treatment in a randomized clinical trial. It is also one of the first worldwide employing GMM models to identify latent subgroups of patients across 1 year follow-up after treatment. Additionally, the study was able to utilize data on a number of psychological mechanistic factors to examine their associations with trajectories of symptom change during therapy for the first time.

However, several limitations to must considered in this study. First, the number of patients in both treatment groups was relatively small, partly due to the proportion of patients that dropped-out during the course of follow-up assessments. Despite this potential concern, we were able to estimate distinct classes through GMM analysis and identify characteristics associated with following different trajectories. Although the rate of drop-out during the follow-up period was similar in both treatment groups (Cano-Vindel et al., 2022) and similar to other RCTs in primary care (Bortolotti, Menchetti, Bellini, Montaguti, & Berardi, 2008). Second, the statistical power of the study was adequate for the main clinical variables, but it was insufficient for some cognitive emotional domains (i.e. attentional biases, metacognition. See Supplementary materials, Tables S3 and S4). There were a number of selection biases that might affect generalizability as well. The mean age of the sample was 44 and the vast majority were female (over 80%). While this is similar to large randomized controlled trials and meta-analyses of treatments for depression (Buckman et al., 2022; Cipriani et al., 2018) it is somewhat un-representative of the clinical population with depression in primary care settings in Spain (King et al., 2008; National Statistics Institute, 2021).

## Conclusions

The findings from this study might be used to consider the potential outcomes of usual care or transdiagnostic CBT in addition to usual care for patients seeking treatment for depression, anxiety, or somatization in primary care. For example, patients with lower severity PHQ-9 scores pre-treatment, might be considered likely to have better treatment outcomes than patients with higher baseline severity and comorbid generalized anxiety, but the reduction of symptoms will likely be larger if they receive TAU + TDG-CBT than TAU alone. This knowledge could guide the clinicians and patients to understand that associated likelihood related with each trajectory in both treatments.

Additionally, identifying specific patterns of treatment response could enable more proactive interventions, such as early adjustment of treatment for those showing signs of deterioration or lack of progress. This adaptive intervention approach could not only improve clinical outcomes but also reduce inefficient use of resources by focusing more intensive treatments on patients who truly need them.

## Supporting information

Prieto-Vila et al. supplementary material Prieto-Vila et al. supplementary material

## Supplementary material

The supplementary material for this article can be found at https://doi.org/10.1017/S0033291724002976.
