# Supplementary material for: Long-term depressive symptoms trajectories following CBT delivered in primary care compared to usual treatment
Source: Psychol Med. 2024 Dec;54(16):4918–27. doi: 10.1017/S0033291724002976 (PMC11779549; doi:10.1017/S0033291724002976)

**Supplementary Tables 1A-E.** *Results of Growth Mixture Modelling analysis per slope in TAU group.*

**Supplementary Table 1A.** *Results of Growth Mixture Modelling quadratic slope with variance fixed to zero in TAU group.*

| Class Solution | Log-Likelihood | H0scaling | AIC | BIC | Adj-BIC | VLMR-LRT p-value | Entropy | Classification  (% per profile) |
| --- | --- | --- | --- | --- | --- | --- | --- | --- |
| **2-class** | **-2931.935** | **1.1489** | **5889.870** | **5934.621** | **5893.419** | **0.0002** | **0.768** | **165/66** |
| 3-class | -2924.393 | 1.1984 | 5882.787 | 5941.308 | 5887.427 | 0.3559 | 0.772 | 62/11/158 |
| 4-class | -2915.584 | 1.2082 | 5873.168 | 5945.459 | 5878.901 | 0.2452 | 0.727 | 29/51/19/132 |
| 5-class | -2910.593 | 1.0711 | 5871.185 | 5957.246 | 5878.010 | 0.1547 | 0.746 | 2/53/17/125/34 |
| 6-class | -2907.352 | 0.9825 | 5872.704 | 5972.535 | 5880.621 | 0.1646 | 0.731 | 11/116/53/30/3/18 |

*Abbreviations*: *AIC* = Akaike information criterion; *BIC* = Bayesian information criterion; *Adj-BIC* = sample size-adjusted Bayesian information criterion; *VLMR-LRT* = Vuong-Lo-Mendell-Rubin likelihood ratio test.

**Supplementary Table 1B.** *Results of Growth Mixture Modelling quadratic slope in TAU group.*

| Class Solution | Log-Likelihood | H0scaling | AIC | BIC | Adj-BIC | VLMR-LRT p-value | Entropy | Classification  (% per profile) |
| --- | --- | --- | --- | --- | --- | --- | --- | --- |
| **2-class** | **-2926.626** | **1.1806** | **5889.252** | **5951.215** | **5894.165** | **0.0878** | **0.759** | **70/161** |
| 3-class | -2919.764 | 1.2608 | 5883.527 | 5959.261 | 5889.533 | 0.5177 | 0.681 | 105/71/55 |
| 4-class | -2913.557 | 1.1872 | 5879.115 | 5968.618 | 5886.212 | 0.3347 | 0.729 | 131/17/55/28 |
| 5-class | -2908.590 | 1.0611 | 5877.180 | 5980.453 | 5885.370 | 0.1864 | 0.756 | 3/31/125/56/16 |
| 6-class | -2900.872 | 1.1280 | 5869.744 | 5986.786 | 5879.025 | 0.4758 | 0.782 | 50/94/15/6/14/52 |

*Abbreviations*: *AIC* = Akaike information criterion; *BIC* = Bayesian information criterion; *Adj-BIC* = sample size-adjusted Bayesian information criterion; *VLMR-LRT* = Vuong-Lo-Mendell-Rubin likelihood ratio test.

**Supplementary Table 1C.** *Results of Growth Mixture Modelling linear slope in TAU group.*

| Class Solution | Log-Likelihood | H0scaling | AIC | BIC | Adj-BIC | VLMR-LRT p-value | Entropy | Classification  (% per profile) |
| --- | --- | --- | --- | --- | --- | --- | --- | --- |
| 2-class | -2956.694 | 1.1066 | 5939.387 | 5984.139 | 5942.936 | 0.0009 | 0.772 | 71/160 |
| **3-class** | **-2950.997** | **1.0759** | **5933.995** | **5989.073** | **5938.362** | **0.1247** | **0.715** | **68/31/131** |
| 4-class | -2948.508 | 1.0364 | 5935.016 | 6000.422 | 5940.203 | 0.3358 | 0.672 | 110/65/20/36 |
| 5-class | -2942.962 | 1.1919 | 5929.924 | 6005.657 | 5935.930 | 0.5848 | 0.783 | 54/36/80/30/31 |
| 6-class | -2939.666 | 1.3203 | 5929.332 | 6015.392 | 5936.156 | 0.7358 | 0.757 | 18/30/33/62/35/53 |

*Abbreviations*: *AIC* = Akaike information criterion; *BIC* = Bayesian information criterion; *Adj-BIC* = sample size-adjusted Bayesian information criterion; *VLMR-LRT* = Vuong-Lo-Mendell-Rubin likelihood ratio test.

**Supplementary Table 1D.** *Results of Growth Mixture Modelling linear slope with variance fixed to zero in TAU group.*

| Class Solution | Log-Likelihood | H0scaling | AIC | BIC | Adj-BIC | VLMR-LRT p-value | Entropy | Classification  (% per profile) |
| --- | --- | --- | --- | --- | --- | --- | --- | --- |
| 2-class | -2942.244 | 1.1165 | 5912.489 | 5960.683 | 5916.311 | 0.0000 | 0.823 | 160/71 |
| 3-class | -2930.472 | 1.2217 | 5894.943 | 5953.465 | 5899.584 | 0.1582 | 0.683 | 65/114/52 |
| 4-class | -2923.833 | 1.1635 | 5887.666 | 5956.514 | 5893.125 | 0.1610 | 0.718 | 22/62/101/46 |
| 5-class | -2918.816 | 1.1662 | 5883.632 | 5962.807 | 5889.910 | 0.3627 | 0.729 | 42/34/22/70/63 |
| 6-class | -2915.103 | 1.1638 | 5882.205 | 5971.708 | 5889.303 | 0.4163 | 0.738 | 65/56/25/20/35/30 |

*Abbreviations*: *AIC* = Akaike information criterion; *BIC* = Bayesian information criterion; *Adj-BIC* = sample size-adjusted Bayesian information criterion; *VLMR-LRT* = Vuong-Lo-Mendell-Rubin likelihood ratio test.

**Supplementary Table 1E.** *Results of Growth Mixture Modelling free loading slope in TAU group.*

| Class Solution | Log-Likelihood | H0scaling | AIC | BIC | Adj-BIC | VLMR-LRT p-value | Entropy | Classification  (% per profile) |
| --- | --- | --- | --- | --- | --- | --- | --- | --- |
| **2-class** | **-2933.448** | **1.2467** | **5898.896** | **5953.975** | **5903.264** | **0.1274** | **0.744** | **161/70** |
| 3-class | -2926.729 | 1.2368 | 5891.458 | 5956.864 | 5896.644 | 0.2557 | 0.689 | 109/70/52 |
| 4-class | -2920.746 | 1.2267 | 5885.493 | 5961.226 | 5891.498 | 0.3262 | 0.752 | 105/12/62/52 |
| 5-class | -2916.731 | 1.1860 | 5883.461 | 5969.522 | 5890.286 | 0.3497 | 0.763 | 33/38/81/45/34 |
| 6-class | -2910.948 | 1.1980 | 5877.896 | 5974.284 | 5885.540 | 0.3583 | 0.777 | 38/30/50/38/35/40 |

*Abbreviations*: *AIC* = Akaike information criterion; *BIC* = Bayesian information criterion; *Adj-BIC* = sample size-adjusted Bayesian information criterion; *VLMR-LRT* = Vuong-Lo-Mendell-Rubin likelihood ratio test.

**Supplementary Tables 2A-E.** *Results of Growth Mixture Modelling analysis per slope in TAU+TDG-CBT group.*

**Supplementary Table 2A.** *Results of Growth Mixture Modelling quadratic with variance fixed to zero in TAU+TDG-CBT group.*

| Class Solution | Log-Likelihood | H0scaling | AIC | BIC | Adj-BIC | VLMR-LRT p-value | Entropy | Classification  (% per profile) |
| --- | --- | --- | --- | --- | --- | --- | --- | --- |
| 2-class | -3201.948 | 1.7812 | 6429.896 | 6475.778 | 6434.566 | 0.1943 | 0.833 | 35/217 |
| 3-class | -3186.959 | 1.4718 | 6407.917 | 6467.918 | 6414.025 | 0.1066 | 0.788 | 176/13/63 |
| **4-class** | **-3173.847** | **1.3983** | **6389.695** | **6463.813** | **6397.240** | **0.1996** | **0.796** | **15/12/176/49** |
| 5-class | -3164.662 | 1.3715 | 6379.324 | 6467.560 | 6388.306 | 0.3905 | 0.760 | 16/25/12/126/73 |
| 6-class | -3158.765 | 1.2222 | 6375.531 | 6477.884 | 6385.950 | 0.4117 | 0.788 | 124/75/1/16/25/11 |

*Abbreviations*: *AIC* = Akaike information criterion; *BIC* = Bayesian information criterion; *Adj-BIC* = sample size-adjusted Bayesian information criterion; *VLMR-LRT* = Vuong-Lo-Mendell-Rubin likelihood ratio test.

**Supplementary Table 2B.** *Results of Growth Mixture Modelling quadratic slope in TAU+TDG-CBT group.*

| Class Solution | Log-Likelihood | H0scaling | AIC | BIC | Adj-BIC | VLMR-LRT p-value | Entropy | Classification  (% per profile) |
| --- | --- | --- | --- | --- | --- | --- | --- | --- |
| 2-class | -3190.674 | 1.4446 | 6417.349 | 6480.878 | 6423.816 | 0.0579 | 0.826 | 38/214 |
| 3-class | -3180.162 | 1.3737 | 6404.323 | 6481.971 | 6412.227 | 0.1614 | 0.841 | 15/200/37 |
| **4-class** | **-3163.720** | **1.3234** | **6379.440** | **6471.206** | **6388.782** | **0.0900** | **0.827** | **17/12/44/179** |
| 5-class | -3157.190 | 1.1625 | 6374.381 | 6480.264 | 6385.159 | 0.0354 | 0.854 | 1/178/10/46/17 |
| 6-class | -3151.025 | 1.2244 | 6370.050 | 6490.050 | 6382.265 | 0.6392 | 0.849 | 18/1/44/174/11/4 |

*Abbreviations*: *AIC* = Akaike information criterion; *BIC* = Bayesian information criterion; *Adj-BIC* = sample size-adjusted Bayesian information criterion; *VLMR-LRT* = Vuong-Lo-Mendell-Rubin likelihood ratio test.

**Supplementary Table 2C.** *Results of Growth Mixture Modelling free loading slope in TAU+TDG-CBT group.*

| Class Solution | Log-Likelihood | H0scaling | AIC | BIC | Adj-BIC | VLMR-LRT p-value | Entropy | Classification  (% per profile) |
| --- | --- | --- | --- | --- | --- | --- | --- | --- |
| 2-class | -3160.237 | 1.5941 | 6352.474 | 6408.945 | 6358.222 | 0.3352 | 0.813 | 214/38 |
| **3-class** | **-3145.240** | **1.2240** | **6328.479** | **6395.539** | **6335.306** | **0.0017** | **0.840** | **172/10/70** |
| 4-class | -3139.536 | 1.2367 | 6323.072 | 6400.719 | 6330.976 | 0.3298 | 0.805 | 10/36/108/98 |
| 5-class | -3132.033 | 1.1345 | 6314.066 | 6402.302 | 6323.048 | 0.0186 | 0.776 | 10/114/59/38/31 |
| 6-class | -3128.271 | 1.1534 | 6312.542 | 6411.366 | 6322.602 | 0.3806 | 0.738 | 39/36/10/29/64/74 |

*Abbreviations*: *AIC* = Akaike information criterion; *BIC* = Bayesian information criterion; *Adj-BIC* = sample size-adjusted Bayesian information criterion; *VLMR-LRT* = Vuong-Lo-Mendell-Rubin likelihood ratio test.

**Supplementary Table 2D.** *Results of Growth Mixture Modelling linear slope in TAU+TDG-CBT group.*

| Class Solution | Log-Likelihood | H0scaling | AIC | BIC | Adj-BIC | VLMR-LRT p-value | Entropy | Classification  (% per profile) |
| --- | --- | --- | --- | --- | --- | --- | --- | --- |
| 2-class | -3264.594 | 1.4474 | 6555.187 | 6601.070 | 6559.858 | 0.0298 | 0.838 | 28/224 |
| 3-class | -3253.308 | 1.3089 | 6538.617 | 6595.088 | 6544.365 | 0.0944 | 0.820 | 13/66/173 |
| 4-class | -3248.005 | 1.2852 | 6534.011 | 6601.070 | 6540.837 | 0.3181 | 0.823 | 13/179/7/53 |
| **5-class** | **-3219.765** | **1.2587** | **6485.531** | **6566.708** | **6493.794** | **0.0589** | **0.750** | **9/38/43/29/133** |
| 6-class | -3236.531 | 1.1500 | 6523.062 | 6611.298 | 6532.044 | 0.1875 | 0.830 | 9/4/43/5/108/83 |

*Abbreviations*: *AIC* = Akaike information criterion; *BIC* = Bayesian information criterion; *Adj-BIC* = sample size-adjusted Bayesian information criterion; *VLMR-LRT* = Vuong-Lo-Mendell-Rubin likelihood ratio test.

**Supplementary Table 2E.** *Results of Growth Mixture Modelling linear slope with variance fixed to zero in TAU+TDG-CBT group.*

| Class Solution | Log-Likelihood | H0scaling | AIC | BIC | Adj-BIC | VLMR-LRT p-value | Entropy | Classification  (% per profile) |
| --- | --- | --- | --- | --- | --- | --- | --- | --- |
| 2-class | -3263.967 | 1.4420 | 6555.933 | 6605.345 | 6560.963 | 0.0004 | 0.862 | 207/45 |
| 3-class | -3239.083 | 1.3906 | 6512.166 | 6572.166 | 6518.274 | 0.0245 | 0.826 | 10/170/72 |
| 4-class | -3228.579 | 1.3136 | 6497.159 | 6567.747 | 6504.344 | 0.1305 | 0.780 | 89/30/10/123 |
| **5-class** | **-3219.765** | **1.2587** | **6485.531** | **6566.708** | **6493.794** | **0.0589** | **0.750** | **9/38/43/29/133** |
| 6-class | -3216.143 | 1.1646 | 6484.287 | 6576.052 | 6493.628 | 0.1355 | 0.775 | 38/132/4/9/43/26 |

*Abbreviations*: *AIC* = Akaike information criterion; *BIC* = Bayesian information criterion; *Adj-BIC* = sample size-adjusted Bayesian information criterion; *VLMR-LRT* = Vuong-Lo-Mendell-Rubin likelihood ratio test.

**Supplementary Table 3.** *Descriptive statistics* TAU.

|  | Total Sample  (n = 231) | Improvers  (n = 165) | No improvers  (n = 66) | *p* value  (*t-test* or χ2) | Statistical power |
| --- | --- | --- | --- | --- | --- |
|  | Mean (*SD*) | Mean (*SD*) | Mean (*SD*) |  |  |
| Age | 45 (11.72) | 44.7 (11.56) | 45.77 (12.17) | .539 | .96 |
| PHQ-9 | 14.12 (4.97) | 12.92 (4.69) | 17,12 (4.35) | <.001 | 1 |
| PHQ-15 | 14.62 (4.45) | 14 (4.33) | 16.17 (4.9) | <.001 | .93 |
| GAD-7 | 12.47 (4.35) | 11.58 (4.28) | 14.7 (3.7) | <.001 | 1 |
| SDS | 23.48 (9.47) | 22.38 (9.43) | 26.23 (9.08) | .005 | .81 |
| WHOQOOL-BREF | 2.89 (.78) | 3.04 (.77) | 2.52 (.68) | <.001 | .99 |
| PSWQ-A | 30.15 (6.5) | 29.23 (6.37) | 32.44 (6.31) | <.001 | .93 |
| RRS brooding | 13.21 (3.55) | 12.58 (3.34) | 14.8 (3.57) | <.001 | .99 |
| IACTA Brief | 7.96 (5.13) | 7.56 (4.94) | 8.97 (5.5) | .076 | .47 |
| ERQ suppression | 15.24 (6.04) | 14.32 (5.94) | 17.53 (5.71) | <.001 | .96 |
| ERQ reinterpretation | 24.77 (6.76) | 24.52 (7.2) | 25.38 (5.59) | .339 | .38 |
| MCQ negative beliefs | 15.84 (4.03) | 15.52 (3.97) | 16.64 (4.09) | .063 | .48 |
| Social support | 4.73 (3.12) | 4.87 (3.1) |  | .443 | .9 |
| Sleeping problems | . (.) | 1.79 (.78) | 2.18 (.94) | .0.06 | .77 |
| Anhedonia | . (.) | 1.71 (.72) | 2.09 (.82) | .0.04 | .83 |
|  | n (%) | n (%) | n (%) |  |  |
| Gender |  |  |  | .82 |  |
| Female | 194 (84) | 138 (71.1) | 56 (28.9) |  |  |
| Male | 37 (16) | 27 (73) | 10 (27) |  |  |
| Marital status |  |  |  | .054 |  |
| With partner | 158 (68.4) | 119 (75.3) | 39 (24.7) |  |  |
| Without partner | 73 (31.6) | 46 (63) | 27 (37) |  |  |
| Educational level |  |  |  | .34 |  |
| Basic studies | 172 (74.5) | 120 (69.8) | 52 (30.2) |  |  |
| High studies | 59 (25.5) | 45 (76.3) | 14 (23.7) |  |  |
| Employment status |  |  |  | .382 |  |
| Employed | 119 (51.5) | 82 (68.9) | 37 (31.1) |  |  |
| Unemployed | 112 (48.5) | 83 (74.1) | 29 (25.9) |  |  |
| Antidepressant use |  |  |  | .009 |  |
| No | 168 (72.7) | 128 (76.2) | 40 (23.8) |  |  |
| Yes | 63 (27.3) | 37 (58.7) | 26 (41.3) |  |  |
| Anxiolytic use |  |  |  | .336 |  |
| No | 151 (65.4) | 111 (73.5) | 40 (26.5) |  |  |
| Yes | 80 (34.6) | 54 (67.5) | 26 (41.3) |  |  |
| PHQ-PD |  |  |  | .063 |  |
| Absence | 72.3 (72.3) | 125 (74.9) | 42 (25.1) |  |  |
| Presence | 64 (27.7) | 40 (62.5) | 24 (37.5) |  |  |
| Suicidal thoughts |  |  |  | .002 |  |
| Absence | 134 | 106 (79.1) | 28 (20.9) |  |  |
| Presence | 97 | 59 (60.8) | 38 (39.2) |  |  |

*Abbreviations*: *SD* = Standard Deviation; *PHQ-9* = Patient Health Questionnaire-9; *PHQ-15* = Patient Health Questionnaire-15; *GAD-7* = Generalized Anxiety Disorder-7; *PHQ-PD* = Patient Health Questionnaire-Panic Disorder; *WHOQOL* = World Health Organization Quality of Life; *SDS* = Sheehan Disability Scale; *PSWQ* = Penn State Worry Questionnaire; *RRS* = Rumination Response Scale; *IACTA* = Inventory of Cognitive Activity in Anxiety Disorders; *ERQ* = Emotional Regulation Questionnaire; *MCQ* = Metacognition Questionnaire; *TAU* = Treatment as usual; *TDG-CBT* = Transdiagnostic group cognitive-behavioral therapy.

**Supplementary Table 4.** *Descriptive statistics TAU+TDG-CBT*

|  | Total sample  (n = 252) | Recovery  (n = 176) | Relapse  (n = 49) | Late recovery (n = 15) | Chronicity  (n = 10) | *p* value (*ANOVA*) | Statistical power |
| --- | --- | --- | --- | --- | --- | --- | --- |
|  | Mean (*SD*) | Mean (*SD*) | Mean (*SD*) | Mean (*SD*) | Mean (*SD*) |  |  |
| Age | 44.4 (10.81) | 43.92 (11.08) | 44.65 (10.84) | 43.87 (8.81) | 51.17 (6.87) | .164 | 1 |
| PHQ-9 | 14.14 (4.95) | 13.05 (4.58) | 15.63 (4.96) | 17.33 (4.65) | 20.08 (2.99) | <.001 | 1 |
| PHQ-15 | 14.27 (4.77) | 13.27 (4.52) | 15.61 (4.3) | 16.93 (4.96) | 20 (3.57) | <.001 | 1 |
| GAD-7 | 12.86 (4.47) | 12.11 (4.41) | 13.82 (4.14) | 15.67 (2.28) | 16.33 (3.45) | <.001 | .98 |
| SDS | 24.23 (9.11) | 23.33 (8.91) | 25.18 (8.49) | 28.87 (12.21) | 27.83 (8.14) | .045 | .66 |
| WHOQOOL-BREF | 2.87 (.81) | 2.97 (.84) | 2.73 (.64) | 2.73 (.88) | 2.17 (.39) | .003 | .9 |
| PSWQ-A | 29.85 (6.84) | 28.89 (6.71) | 30.98 (7.15) | 33.33 (5.7) | 35 (4.65) | <.001 | .93 |
| RRS brooding | 13.49 (3.57) | 12.89 (3.38) | 14.39 (3.59) | 14.8 (3.89) | 16.83 (3.04) | <.001 | .98 |
| IACTA Brief | 8.75 (5.21) | 8.39 (5.17) | 9.49 (5.12) | 8.8 (5.91) | 10.75 (5.12) | .314 | .32 |
| ERQ suppression | 15.74 (5.76) | 15.54 (5.82) | 16.65 (5.38) | 14.2 (5.17) | 17 (7.05) | .38 | .28 |
| ERQ reinterpretation | 25.89 (6.96) | 26.13 (6.99) | 24.59 (6.79) | 25 (6.99) | 28.75 (6.73) | .242 | .37 |
| MCQ negative beliefs | 16.64 (3.96) | 16.31 (3.87) | 17.08 (3.91) | 18.07 (4.27) | 17.91 (4.58) | .167 | .44 |
| Sleeping problems | 1.9 (.98) | 1.81 (.94) | 2.06 (1.05) | 2.13 (.99) | 2.33 (1.15) | .105 | .53 |
| Anhedonia | 1.74 (.93) | 1.64 (.95) | 1.9 (.82) | 1.87 (.99) | 2.33 (.78) | .036 | .68 |
|  | n (%) | n (%) | n (%) | n (%) | n (%) | *p* value (χ2) |  |
| Gender |  |  |  |  |  | .482 |  |
| Female | 199 (79) | 135 (67.8) | 40 (20.1) | 14 (7) | 10 (5) |  |  |
| Male | 53 (21) | 41 (77.4) | 9 (17) | 1 (1.9) | 2 (3.8) |  |  |
| Marital status |  |  |  |  |  | .381 |  |
| With partner | 181 (71.8) | 127 (70.2) | 37 (20.4) | 11 (6.1) | 6 (3.3) |  |  |
| Without partner | 71 (28.2) | 49 (69) | 12 (16.4) | 4 (5.6) | 6 (8.5) |  |  |
| Educational level |  |  |  |  |  | .604 |  |
| Basic studies | 173 (68.7) | 116 (67.1) | 37 (21.4) | 11 (6.4) | 9 (5.2) |  |  |
| High studies | 79 (31.3) | 60 (75.9) | 12 (15.2) | 4 (5.1) | 3 (3.8) |  |  |
| Employment status |  |  |  |  |  | .379 |  |
| Employed | 136 (54) | 94 (69.1) | 30 (22.1) | 8 (5.9) | 4 (2.9) |  |  |
| Unemployed | 116 (46) | 82 (70.7) | 19 (16.4) | 7 (6) | 8 (6.9) |  |  |
| Antidepressant use |  |  |  |  |  | <.001 |  |
| No | 196 (77.8) | 148 (75.5) | 36 (18.4) | 7 (3.6) | 5 (2.6) |  |  |
| Yes | 56 (22.2) | 28 (50) | 13 (23.2) | 8 (14.3) | 7 (12.5) |  |  |
| Anxiolytic use |  |  |  |  |  | .711 |  |
| No | 151 (59.9) | 108 (71.5) | 26 (17.2) | 10 (6.6) | 7 (4.6) |  |  |
| Yes | 101 (40.1) | 68 (67.3) | 23 (22.8) | 5 (5) | 5 (5) |  |  |
| PHQ-PD |  |  |  |  |  | .103 |  |
| Absence | 184 (73) | 132 (71.7) | 35 (19) | 12 (6.5) | 5 (2.7) |  |  |
| Presence | 68 (27) | 44 (64.7) | 14 (20.6) | 3 (4.4) | 7 (10.3) |  |  |
| Suicidal thoughts |  |  |  |  |  | <.001 |  |
| Absence | 157 (62.3) | 123 (78.3) | 25 (15.9) | 7 (4.5) | 2 (1.3) |  |  |
| Presence | 95 (37.7) | 53 (55.8) | 24 (25.3) | 8 (8.4) | 10 (10.5) |  |  |

*Abbreviations*: *SD* = Standard Deviation; *PHQ-9* = Patient Health Questionnaire-9; *PHQ-15* = Patient Health Questionnaire-15; *GAD-7* = Generalized Anxiety Disorder-7; *PHQ-PD* = Patient Health Questionnaire-Panic Disorder; *WHOQOL* = World Health Organization Quality of Life; *SDS* = Sheehan Disability Scale; *PSWQ* = Penn State Worry Questionnaire; *RRS* = Rumination Response Scale; *IACTA* = Inventory of Cognitive Activity in Anxiety Disorders; *ERQ* = Emotional Regulation Questionnaire; *MCQ* = Metacognition Questionnaire; *TAU* = Treatment as usual; *TDG-CBT* = Transdiagnostic group cognitive-behavioral therapy.

**Supplementary Table 5.** *Associations between baseline characteristics and PHQ-9 trajectory classes 2 – late recovery relative to class 4 - relapse.*

| Baseline predictor | Late recovery  OR (95%CI) & *p*-value |
| --- | --- |
| GAD-7 | 1.156 (.951 - 1.406), p = .146 |
| PHQ-15 | 1.037 (.883 - 1.218), p = .661 |
| Anhedonia | .638 (.269 - 1.51), p = .306 |
| Suicidal thoughts |  |
| Absence | .577 (.172 – 1.929), p = .372 |
| Presence | Ref. |
| SDS | 1.054 (.972 – 1.143), p = .202 |
| WHOQOOL | 1.74 (.638 - 4.745), p = .279 |
| PSWQ | 1.041 (.919 - 1.179), p = .527 |
| RRS | .842 (.222 - 3.189), p = .8 |
| Antidepressant use |  |
| No | .254 (.064 - 1.016), p = .053 |
| Yes | Ref. |

*Abbreviations*: *PHQ-15* = Patient Health Questionnaire-15; *GAD-7* = Generalized Anxiety Disorder-7; *WHOQOL* = World Health Organization Quality of Life; *SDS* = Sheehan Disability Scale; *PSWQ* = Penn State Worry Questionnaire; *RRS* = Rumination Response Scale.

**Supplementary Figure 1.** *Flow-chart of the participants of the original and current study.*


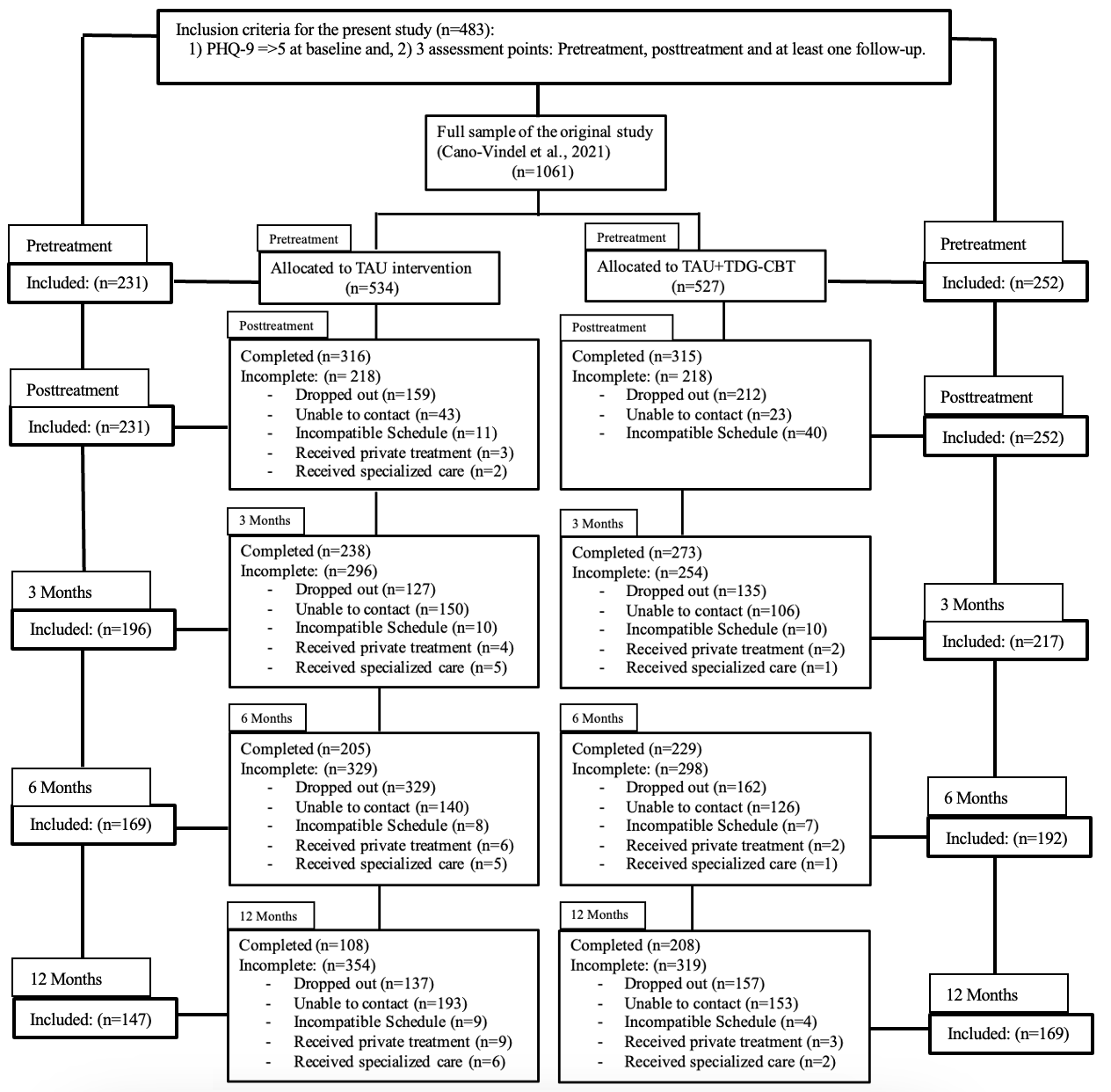

Supplement: Prieto-Vila et al. supplementary material [file S0033291724002976sup001.docx]
